# Evaluation of the operational challenges in implementing reactive screen-and-treat and implications of reactive case detection strategies for malaria elimination in a region of low transmission in southern Zambia

**DOI:** 10.1186/s12936-016-1460-x

**Published:** 2016-08-15

**Authors:** Kelly M. Searle, Harry Hamapumbu, Jailos Lubinda, Timothy M. Shields, Jessie Pinchoff, Tamaki Kobayashi, Jennifer C. Stevenson, Daniel J. Bridges, David A. Larsen, Philip E. Thuma, William J. Moss, for the Southern Africa International Centers of Excellence for Malaria Research

**Affiliations:** 1 Department of Epidemiology, Bloomberg School of Public Health, Johns Hopkins University, Baltimore, MD USA; 2 Johns Hopkins Malaria Research Institute, Department of Molecular Microbiology and Immunology, Bloomberg School of Public Health, Johns Hopkins University, Baltimore, MD USA; 3 Macha Research Trust, Choma District, Zambia; 4 Akros, Cresta Golfview Grounds, Great East Road, Lusaka, Zambia; 5 Department of Public Health, Food Studies and Nutrition, Syracuse University, Syracuse, NY USA

**Keywords:** Malaria elimination, Reactive case detection, Reactive test-and-treat, Zambia, Sub-Saharan Africa

## Abstract

**Background:**

As malaria transmission declines in many regions of sub-Saharan Africa, interventions to identify the asymptomatic reservoir are being deployed with the goals of improving surveillance and interrupting transmission. Reactive case detection strategies, in which individuals with clinical malaria are followed up at their home and household residents and neighbours are screened and treated for malaria, are increasingly used as part of malaria elimination programmes.

**Methods:**

A reactive screen-and-treat programme was implemented by the National Malaria Control Centre in Southern Province, Zambia, in which individuals residing within 140 m of an index case were screened with a malaria rapid diagnostic test (RDT) and treated if positive. The operational challenges during the early stages of implementing this reactive screen-and-treat programme in the catchment area of Macha Hospital in Southern Province, Zambia were assessed using rural health centre records, ground truth evaluation of community health worker performance, and data from serial cross-sectional surveys. The proportion of individuals infected with *Plasmodium falciparum* who were identified and treated was estimated by simulating reactive screen-and-treat and focal drug administration cascades.

**Results:**

Within the 1st year of implementation, community health workers followed up 32 % of eligible index cases. When index cases were followed up, 66 % of residents were at home in the index households and 58 % in neighbouring households. Forty-one neighbouring households of 26 index households were screened, but only 13 (32 %) were within the 140-m screening radius. The parasite prevalence by RDT was 22 % in index households and 5 % in neighbouring households. In a simulation model with complete follow-up, 22 % of the total infected population would be detected with reactive screen-and-treat but 57 % with reactive focal drug administration.

**Conclusions:**

With limited resources, coverage and diagnostic tools, reactive screen-and-treat will likely not be sufficient to achieve malaria elimination in this setting. However, high coverage with reactive focal drug administration could be efficient at decreasing the reservoir of infection and should be considered as an alternative strategy.

**Electronic supplementary material:**

The online version of this article (doi:10.1186/s12936-016-1460-x) contains supplementary material, which is available to authorized users.

## Background

Substantial reductions in the burden of malaria have been documented in parts of sub-Saharan Africa and malaria elimination goals have been proposed at regional, national and sub-national levels [1–3]. As areas make the transition from malaria control to elimination, strategies have been developed to target the population of chronically infected individuals who are asymptomatic yet can contribute to transmission [4–9]. In malaria-endemic areas, individuals develop clinical immunity to disease after repeated exposure to parasites but can remain infectious despite the absence of symptoms or develop low-grade symptoms that would not prompt them to seek care [10, 11]. As malaria transmission declines, the proportion of the total infected population comprised of asymptomatic, chronically infected individuals with low parasite densities increases [12–15]. These individuals constitute an asymptomatic reservoir that is less infectious than symptomatic, but are capable of transmitting parasites in areas with competent vectors [12, 14, 15].

Several strategies have been developed to identify and treat asymptomatic, chronically infected individuals. Mass drug administration treats entire populations or high-risk groups based on the fact that current point-of-care diagnostic tests are not sufficiently sensitive to identify individuals with low-level parasitaemia [16, 17]. Active case detection, in contrast, involves screening individuals for malaria with rapid diagnostic tests (RDTs) within a defined geographic area (‘hot spots’) or high-risk populations (‘hot pops’) at regular intervals and treating those who test positive. Active case detection and focal and mass drug administration aim to eliminate parasites from chronically infected individuals, thus facilitating the interruption of local transmission [18]. The World Health Organization recommends that areas with moderate to low malaria transmission implement active case detection as part of national malaria control and elimination programmes [8].

One method of active case detection involves reactive case detection, which leverages the underlying spatial and temporal clustering of malaria [19–21]. Reactive case detection includes reactive screen-and-treat and reactive focal drug administration. For reactive screen-and-treat, residents in the home of a symptomatic index case and those in neighbouring households within a specified distance are screened with an RDT and treated if positive [6, 22, 23]. With reactive focal drug administration, individuals residing within an index case household and potentially neighbouring households are treated with anti-malarials without testing [24, 25]. The advantage of focal drug administration is that infected individuals are treated who may otherwise be missed with a low-sensitivity RDT [24, 25]. Uninfected individuals are also treated but may benefit from chemoprophylaxis [24–26].

The Government of Zambia created a stepped sequence of interventions to achieve malaria elimination [27–29]. Designated as steps A through E, these interventions are to be implemented in succession depending on the parasite prevalence and case burden at health facilities [28, 29]. Step D consists of training volunteer community health workers (CHWs) to perform reactive screen-and-treat. Step D is implemented in low-transmission communities in which the parasite prevalence is approximately 1 % and an average of ten or fewer malaria cases present to a healthcare facility per week [28]. In 2013, Step D activities were implemented through a phased roll-out in selected districts in Southern Province, Zambia with the goals of improving surveillance and interrupting transmission [9, 27].

When an individual seeks care at a healthcare facility (hospital, rural health centre or rural health post) and tests positive for malaria by RDT, their eligibility for follow-up with reactive screen-and-treat is determined. CHWs exclude individuals with a reported travel history as these cases are presumed to be imported. Travel is defined as staying overnight in a place outside their home district within the previous month. RDT-positive individuals who had not travelled are eligible for reactive screen-and-treat. Eligible index cases are to be followed up within 1 week of diagnosis. CHWs are trained to visit the households of eligible index cases and neighbouring households within 140 m of an index case, screen all residents with an RDT and treat everyone who tests positive [9].

The study was conducted in Kalomo, Namwala and Choma Districts in Southern Province, Zambia where the single rainy season lasts from November through April, followed by a cool dry season from April until August and a hot dry season through November. Malaria transmission peaks during the rainy season [30]. This area consists of villages comprised of small, scattered homesteads. The primary malaria vector is *Anopheles arabiensis* [30, 31]. The prevalence of malaria declined over the past decade, from 9 % in 2008 to less than 1 % in 2013 [32], generating interest in malaria elimination. Challenges faced during the 1st year of implementation of a reactive screen-and-treat programme in southern Zambia were evaluated. RDT availability, follow-up and coverage were assessed using rural health centre (RHC) records and evaluation of CHW performance. Additional data from serial cross-sectional surveys were used to construct simulated reactive case detection cascades.

## Methods

### Study population

The study population for the record review and ground truth survey consisted of individuals eligible for reactive screen-and-treat from January to June 2014 as described below. Results from the record review and ground truth evaluation were used in combination with data from serial cross-sectional surveys conducted in the catchment area of Macha Hospital from 2008 to 2013. For these cross-sectional surveys, a random sample of households was selected using satellite imagery every other month from February 2008 to December 2013. For each study visit, informed consent was obtained from participants and a questionnaire was administered to collect information on demographic characteristics, recent malaria symptoms and treatment, health-seeking behaviour, and knowledge of malaria risk and prevention. A blood sample was collected by finger prick and tested by RDT (ICT Malaria P.f, ICT Diagnostics South Africa). Participants who tested positive were offered treatment with artemether–lumefantrine (Coartem^®^) [23, 33].

### Record review of reactive screen-and-treat

The reactive screen-and-treat programme started in the study area in May 2013. During the low-transmission season from July to September 2014, a study team from Macha Research Trust visited ten RHCs in Kalomo, Choma and Namwala Districts and abstracted data on reactive screen-and-treat from January to June 2014 (within the 1st year of step D implementation) from 20 rural health posts (RHPs) serving the catchment areas of the ten RHCs. RHPs are the lowest level of stationary healthcare and are staffed by volunteer CHWs. The number of RDTs received by a RHP, tests performed, RDT-positive malaria cases identified, malaria cases eligible for reactive screen-and-treat, and eligible malaria cases followed up were recorded for each month. Reported reasons why eligible cases were not followed up were documented. Since 2013, healthcare facilities used the SD Bioline Malaria Ag P.f (Standard Diagnostics Inc, Republic of Korea).

### Ground truth evaluation of reactive screen-and-treat

To ground truth reactive screen-and-treat performance, study staff visited ten RHPs associated with seven parent RHCs from July to September 2014 and identified index cases with RDT-confirmed malaria that triggered reactive screen-and-treat. Study staff then randomly selected 26 index case households that were screened during Step D activities between January and June 2014 for ground truth evaluation. The study staff and CHW visited the selected index case households and neighbouring households determined to be eligible for screening by the CHW. The study team recorded the number of residents within each household, the number of residents at home and tested by the CHW, the number of RDT-positive residents, the number of residents treated for malaria, and the distance from the index case household to neighbouring households (using a GPS-enabled device). The time from identification of the index case to reactive screen-and-treat was calculated when dates were available. Data on age and sex were collected retrospectively from RHP records.

### Construction of simulated reactive case detection cascades

The proportion of infected individuals identified and treated through reactive case detection was modelled. First, data collected through serial cross-sectional surveys were used to estimate the number of total residents and *Plasmodium falciparum*-infected residents in index and neighbouring households. Details of the model were previously described [23]. In brief, all households in the study area were enumerated from satellite imagery and data from serial cross-sectional surveys were used to predict the number of residents per non-sampled household and the total number of infected individuals positive for *P. falciparum* by PCR in non-sampled households based on an ecological risk map [23]. Second, the proportion of infected individuals who were symptomatic (i.e., documented tympanic temperature ≥38 °C or self-reported fever in previous 48 h) was estimated using data from the serial cross-sectional surveys and extrapolated to non-sampled households. Third, the proportion of symptomatic, infected individuals who sought care from a healthcare facility during their last febrile episode was estimated using data from the serial cross-sectional surveys and extrapolated to the estimated number of symptomatic-infected individuals in the non-sampled households. Fourth, a sensitivity of 95 % was used to determine the proportion of symptomatic-infected individuals who would be RDT-positive upon presentation to a healthcare facility [34]. Fifth, data from the record review and ground truth evaluation were used to determine the median number of neighbouring households per index household that would be screened as well as the parasite prevalence by RDT in index and neighbouring households. Sixth, the sensitivity of the RDT to detect asymptomatic infection was estimated to be 40 % in index households and 23 % in neighbouring households compared to PCR, based on unpublished data comparing RDT and PCR results in the study area. These sensitivities were used to back-calculate the estimated number of infected individuals in index and neighbouring households. Specifically, the inverse of RDT sensitivities for index (1/0.4) and neighbouring households (1/0.23) were multiplied by the estimated number of RDT-positive individuals to estimate the total number of infected individuals.

This model was used to estimate the proportion of infected individuals identified and treated under observed and complete coverage with reactive screen-and-treat. The model was also used to determine the proportion of infected individuals treated using reactive focal drug administration in the index households only. The sensitivity of the model under varying assumptions, including RDT prevalence in index and neighbouring households, RDT sensitivity, and the ration of symptomatic to asymptomatic cases, was estimated. These results are presented in the Additional files 1, 2, 3.

## Results

### Record review of reactive screen-and-treat

Records reviewed at the ten RHCs indicated that 411 malaria cases were passively identified by RDT from January to June 2014 at the 20 RHPs. Of these, 21 cases were excluded by the CHWs based on reported travel history and 394 were considered eligible for follow-up with reactive screen-and-treat. Of those eligible, 32 % (n = 126) were followed up. The primary reason households were not followed up was insufficient RDTs.

**Fig. 1:**
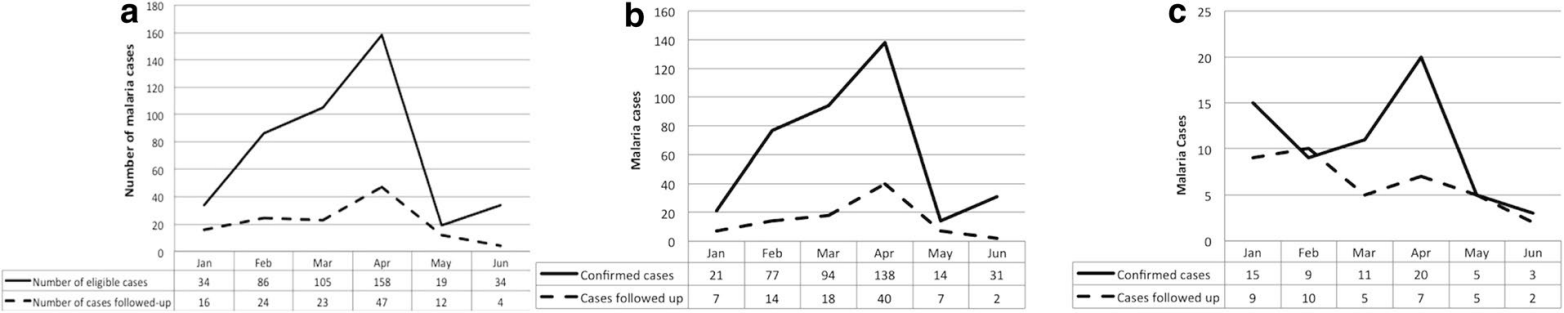
Malaria cases reported and followed up with reactive screen-and-treat by month from record review. **a** All RHPs, **b** high burden RHPs, **c** low burden RHPs

As expected, a seasonal pattern of malaria cases was observed, with the number of cases increasing after January and peaking in April (Fig. 1). As the number of malaria cases increased, the proportion followed up decreased (Fig. 1). When RHPs were stratified by malaria burden (high malaria burden was defined as those with 20 or more eligible cases per month), high-burden RHPs had poorer follow-up as the burden of reactive case detection exceeded capacity (Fig. 1). Over half of the RHPs (n = 11) reported at least 1 month without sufficient RDTs to follow up eligible index cases, and eight of these RHPs reported at least 1 month without sufficient RDTs to perform passive case detection. Low-burden RHPs reported more months with insufficient RDTs than high-burden RHPs. The parent RHCs did not report RDT stock-outs, suggesting challenges in distributing RDTs from RHCs to RHPs.

### Ground truth evaluation of reactive screen-and-treat

**Fig. 2:**
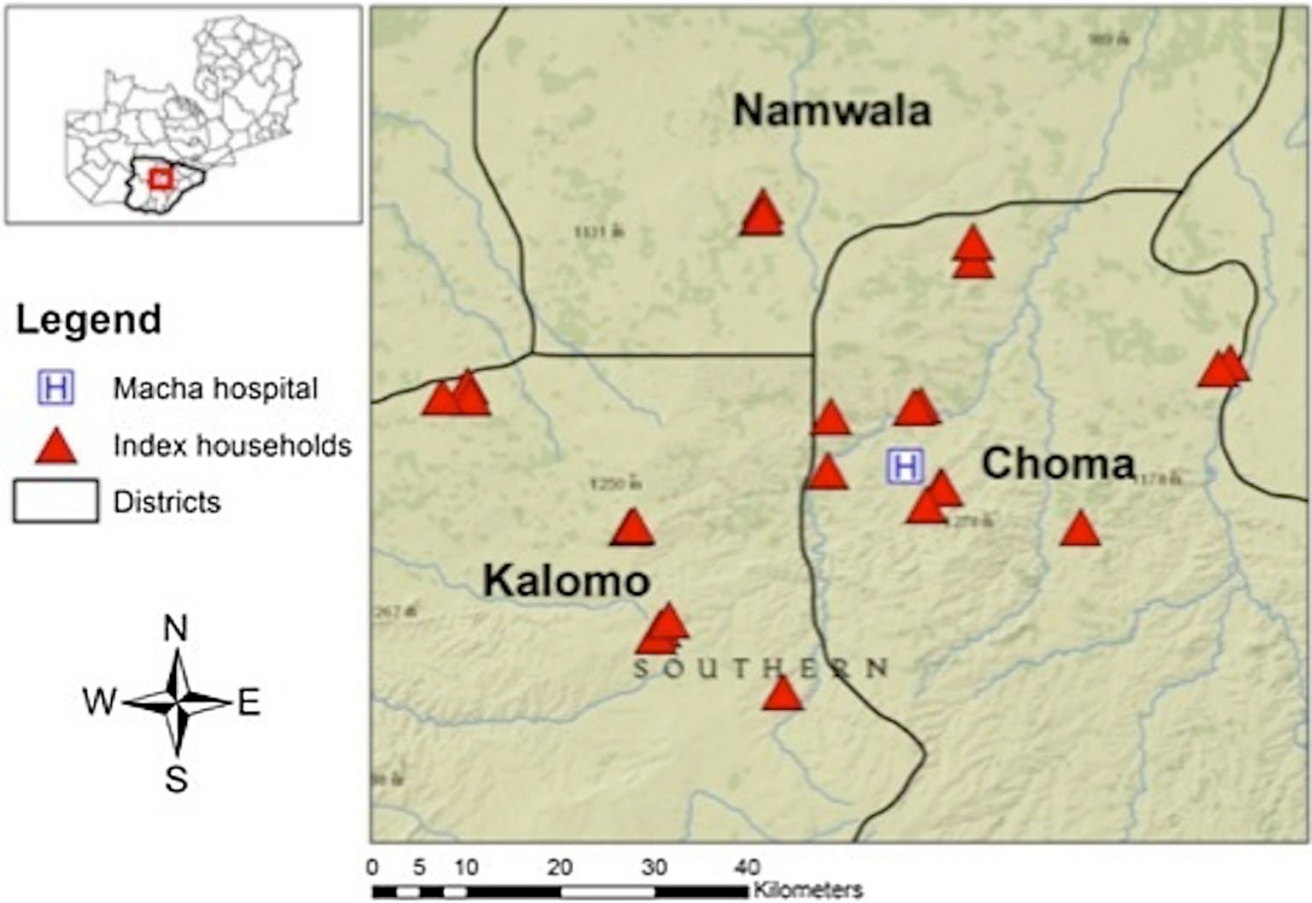
Index households included in the ground-truth evaluation of reactive screen-and-treat

The CHW registers identified 63 neighbouring households associated with the 26 index case households as eligible for reactive screen-and-treat (89 total households) (Table 1). Study staff collected coordinates and household demographics for all 26 index case households and 89 % (n = 56) of the 63 neighbouring households, as no one was home at seven households (Fig. 2). Twenty-two neighbouring households were not screened by CHWs (35 % of 63), of which 12 (55 %) were not screened due to a lack of RDTs, representing 19 % of the 63 eligible households. Of the 41 neighbouring households screened by the CHW, only 13 (32 %) were within 140 m of an index case household (Table 1). Study staff identified 21 households within 140 m of an index household (eight more than the CHWs) and data were collected from 18 (86 %) of these households, as no one was home at three households (Table 1). Thus, the percentage of eligible neighbouring households within 140 m of an index household screened by the CHWs was 62 % (13 of 21) (Table 1). The median number of households screened per index case households was three (IQR 1, 3; minimum = 1; maximum = 8).

**Table 1:** Ground-truth evaluation of reactive screen-and-treat: household and individual characteristics

	Index	Neighbouring	Total
*Household characteristic*			
Indicated eligible for RCD by CHW (%)	26 (100)	63 (100)	89 (100)
Identified by study staff (%)	26 (100)	56 (89)	82 (92)
Recorded in CHW register (%)	26 (100)	41(65)	67 (75)
Within 140 m of index household (%)	NA	21(33)	NA
Within 140 m of index household in CHW register (%)	NA	13 (21)	NA
*Resident characteristic*			
Indicated eligible for RCD by CHW in 82 households with data (%)	261 (100)	444 (100)	705 (100)
Screened and recorded in CHW register (%)	171 (66)	257 (58)	428 (61)
Within 140 m of index household (%)	NA	165 (37)	NA
Within 140 m of index household in CHW register (%)	NA	100 (23)	NA
RDT positive (% of all RDTs)	37 (22)	13 (5)	50 (12)
RDT negative (% of all RDTs)	134 (78)	244 (95)	378 (88)

*RCD* reactive case detection, *CHW* community health worker, *RDT* rapid diagnostic test

For the 26 index cases selected for evaluation, 705 individuals residing in 82 households were eligible for reactive screen-and-treat, 261 in index households and 444 in neighbouring households (Table 1). Overall, 428 individuals (61 %) were recorded to have been screened in the CHW registers. In index case households, 66 % of the residents were reported to be screened compared with 58 % in neighbouring households (p = 0.04) (Table 1). Within the 18 neighbouring households within 140 m for which data were available, 100 (61 %) of 165 eligible individuals were screened by the CHW (Table 1). The parasite prevalence by RDT was 22 % among residents of index case households and 5 % among residents of neighbouring households (Table 1).

The median time between when an index case presented to a healthcare facility and the reactive screen-and-treat was 3 days (IQR = 2–5.5; minimum = 1; maximum = 12). The median distance from the index household to the neighbouring households screened was 194 m (IQR = 117–303; minimum = 36; maximum = 530 m). Thirty-two per cent of all neighbouring households screened by the CHWs were within 140 m of the index case household, suggesting the CHWs had difficulty delineating the 140-m radius. Thirteen RDT-positive individuals were detected in index and neighbouring households. Only one RDT-positive individual (7.7 %) was within 140 ms of the index case household. However, 92 % (n = 12) of all RDT-positive individuals resided within 250 m of the index household (Table 2).

**Table 2:** Cumulative numbers of neighbouring households, individuals, and RDT-positive cases by distance from index households identified through the ground-truth evaluation of reactive screen-and-treat

	140 m	250 m	300 m	350 m	400 m	450 m	500 m	550 m
Households indicated as eligible by CHW (% indicated as eligible)	21 (33)	40 (64)	47 (75)	49 (78)	55 (87)	59 (93)	62 (98)	63 (100)
Households screened by CHW (% households screened)	13 (32)	28 (68)	30 (73)	32 (78)	35 (85)	37 (90)	40 (98)	41 (100)
Residents in screened households (% residents screened)	78 (30)	182 (71)	196 (76)	214 (83)	236 (92)	239 (93)	254 (99)	257 (100)
RDT positive cases in screened households (% RDT positive cases)	1 (8)	12 (92)	12 (92)	13 (100)	13 (100)	13 (100)	13 (100)	13 (100)

*RDT* rapid diagnostic test, *CHW* community health worker

Demographic information was collected from 449 individuals eligible for screening by reactive screen-and-treat, 99 from index case households and 350 from neighbouring households. No overall differences in screening by sex were observed (49.5 % male, 50.7 % female, p = 0.21) or when stratified by household type (index: 41.6 % male, 58.4 % female, p = 0.28; neighbouring: 51.3 % male, 48.7 % female, p = 0.32). Residents of index and neighbouring households did not differ by age (index: median age = 13.5 years IQR = 7–23 years; neighbouring: median age = 14 years IQR = 6–26 years, p = 0.75). However, residents who were screened were younger than those who were not (screened: median age = 13 years, IQR = 6–25 years; not screened: median age = 19 years, IQR = 12–31 years, p < 0.01).

### Reactive case detection cascades

**Fig. 3:**
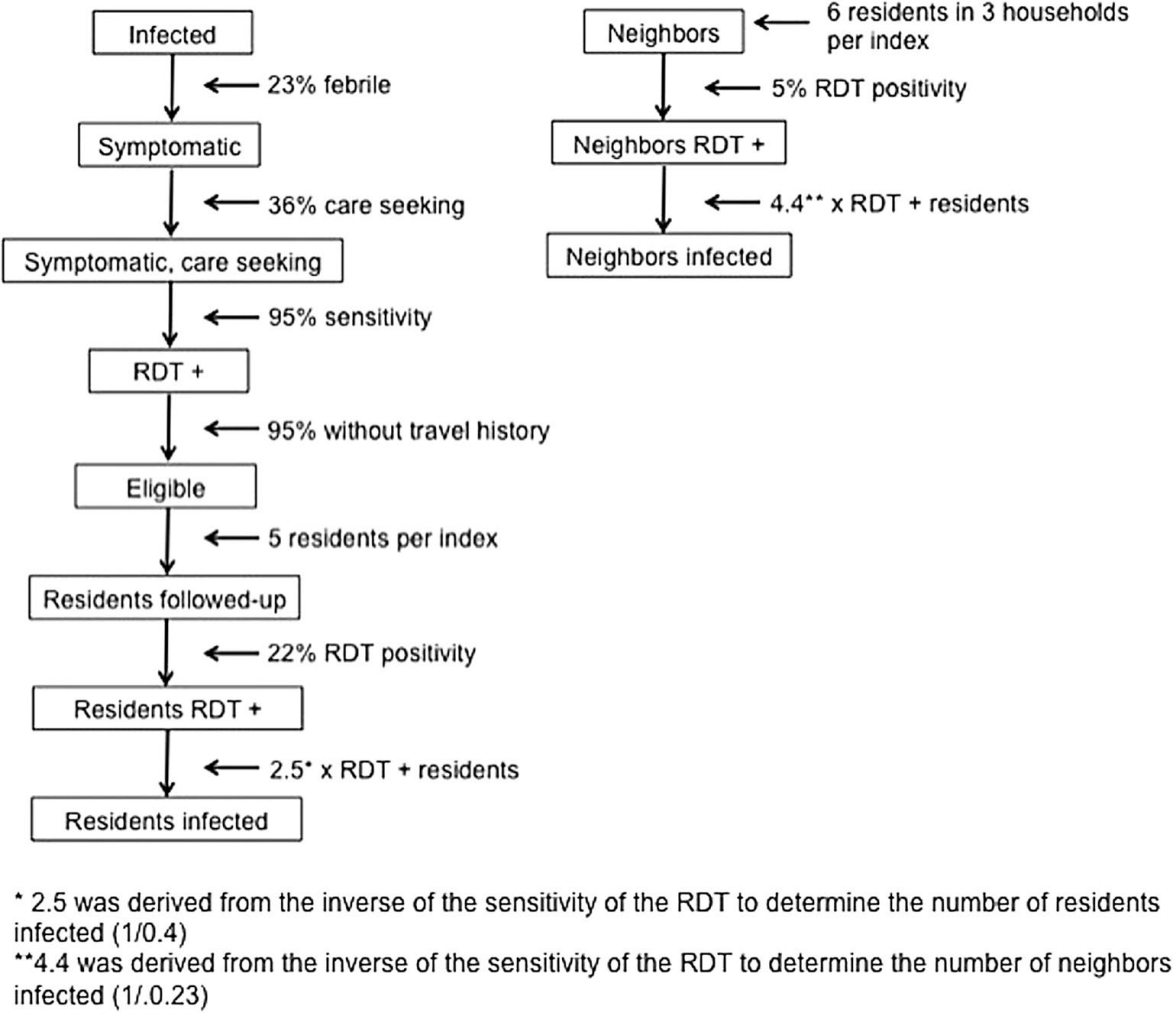
Reactive screen-and-treat flow diagram for complete coverage

A flow diagram of the reactive screen-and-treat and focal drug administration cascade construction and estimates is presented in Fig. 3. The total population of the study area was estimated to be 32,370 individuals and the *P. falciparum* parasite prevalence by PCR was estimated to be 2.9 % (937 infections) [23]. Based on data from the serial cross-sectional surveys, 23 % (n = 214) of the 937 PCR-positive individuals were estimated to be symptomatic (i.e., febrile), with 36 % (n = 76) estimated to seek care at a healthcare facility and thus be potentially eligible to trigger reactive screen-and-treat (Fig. 3). Using an RDT sensitivity of 95 % for uncomplicated malaria, 73 of these individuals would be identified as having malaria at a healthcare facility (Fig. 3) [34]. The proportions of RDT-positive residents in the index and neighbouring households were estimated based on the RDT prevalence observed during the record reviews (22 % in index households and 5 % in neighbouring households) to capture the local spatial dependence of malaria transmission (Fig. 3).

**Fig. 4:**
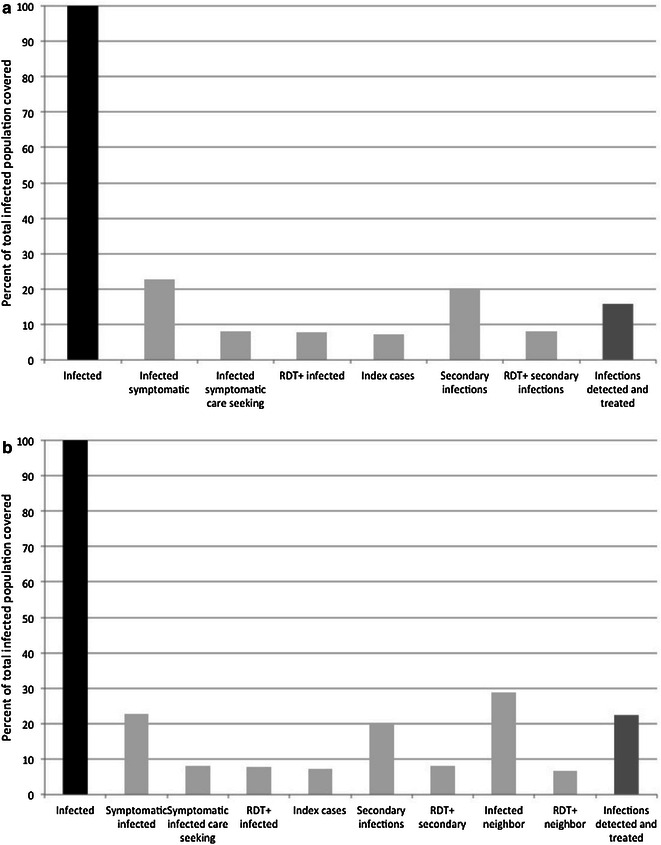
Coverage cascades of reactive screen-and-treat with complete coverage of index and neighbouring households. **a** Index households only, **b** index households and neighbors

**Fig. 5:**
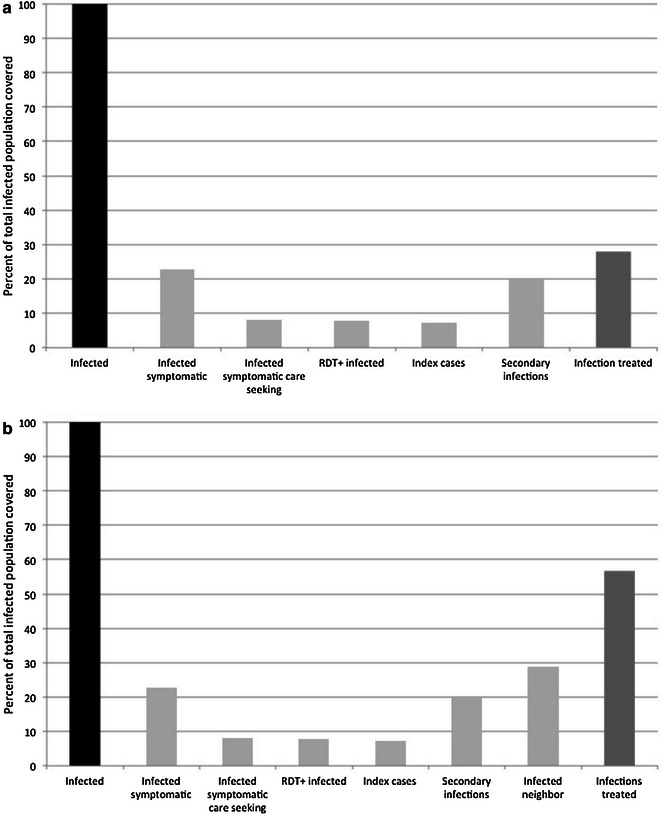
Coverage cascades of reactive focal drug administration with complete coverage of index and neighbouring households. **a** index households only, **b** index households and neighbors

**Fig. 6:**
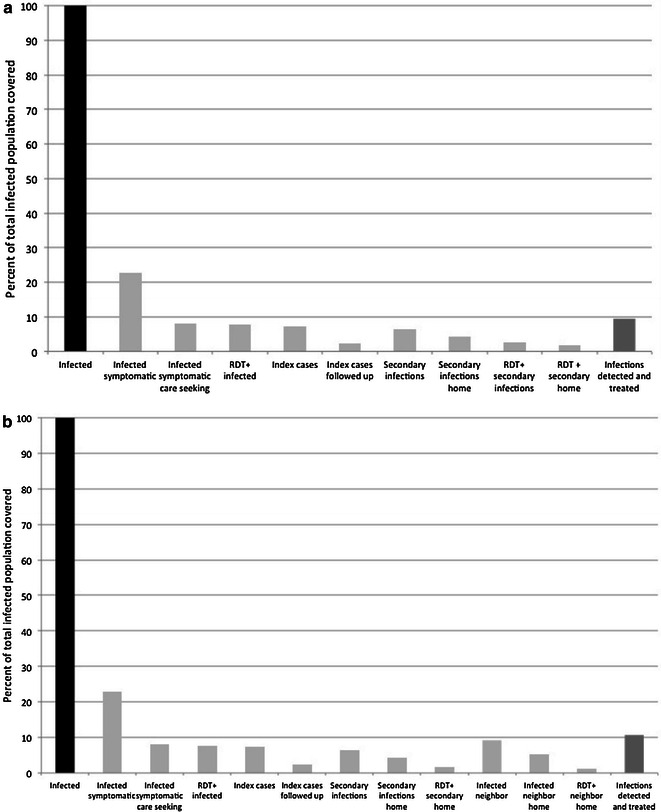
Coverage cascades of reactive screen-and-treat with observed coverage of index and neighbouring households. a Index households only, b index households and neighbors. **a** Index households only, **b** index households and neighbors

**Fig. 7:**
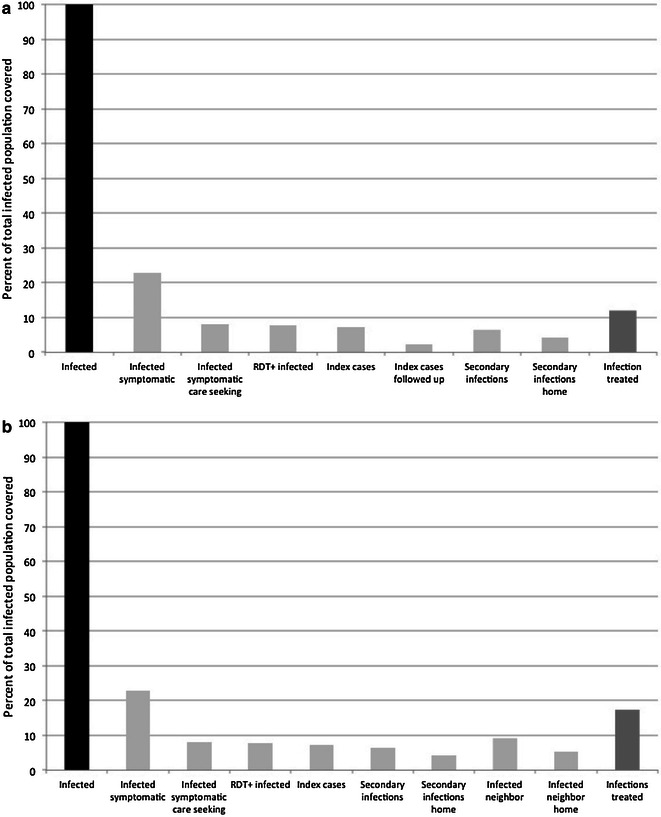
Coverage cascades of reactive focal drug administration with observed coverage of index and neighbouring households. **a** Index households only, **b** index households and neighbors

Based on the eligibility criteria in which persons with recent travel are excluded, 5 % of RDT-positive symptomatic cases were estimated to be ineligible for reactive screen-and-treat (Figs. 4, 5, 6, 7). Thus, 95 % (n = 69) of the RDT-positive index cases were estimated to be eligible for reactive screen-and-treat. These cases represented 7 % (69 of 937) of the total infected population (Figs. 4a, 5, 6, 7a). Based on estimates of household size, an average of five household residents would be screened within each of the 69 index households, yielding 345 residents, with 22 % (n = 76) estimated to be RDT positive (Fig. 4a). Given a 40 % sensitivity of the RDT in index households, 189 infected individuals (20 % of total infections) were estimated to reside within the index households (Fig. 4a) [35]. With complete follow-up, in which all index household residents were screened, 16 % (73 symptomatic RDT-positive index cases and 76 RDT-positive individuals residing within the index case household) of all infected individuals would be detected and treated through reactive screen-and-treat in the index households (Fig. 4a).

When neighbouring households were included in the model, 270 infected individuals were estimated to reside in the 207 neighbouring households of the 69 index households (Fig. 4b), representing 29 % of all infected individuals. Of these infected individuals, 62 (7 % of all infected individuals) were estimated to be RDT-positive based on an RDT sensitivity of 23 %. Screening neighbouring households of the index case household would increase the percentage of the total infected population detected and treated from 16 to 22 %, assuming complete follow-up of all eligible index cases and neighbouring households, and with all residents at home and willing to be screened (Fig. 4b).

Using the same data consisting of 69 index cases, 189 infected individuals in index households, and 270 infected individuals in neighbouring households, the effectiveness of reactive focal drug administration was simulated. In this analysis, the sensitivity of the RDT for asymptomatic infected individuals is not relevant as residents are treated without testing. With complete follow-up of all eligible index cases, 28 % (n = 258), of all infected individuals would be treated through focal drug administration at the index household (Fig. 5a). When neighbouring households were included, 57 % (n = 531) of all infected individuals would be treated through focal drug administration (Fig. 5b).

These data were also used to model the proportion of infected individuals treated under the coverage observed during the RHC evaluation and ground truth surveys, in which follow-up of eligible index cases was only 32 %. In index households, 66 % of the residents were at home during screening and 58 % were at home in neighbouring households. Under observed coverage, screening and treating index case households would have detected and treated only 9 % of the total infected population (Fig. 6a), which increased to 11 % of the total infected population when neighbouring households were included (Fig. 6b). The same observed coverage was used to model focal drug administration. Under observed coverage, 11 % of all infected individuals in the population would have been treated (Fig. 7a), which increased to 17 % when neighbouring households were included (Fig. 7b).

**Fig. 8:**
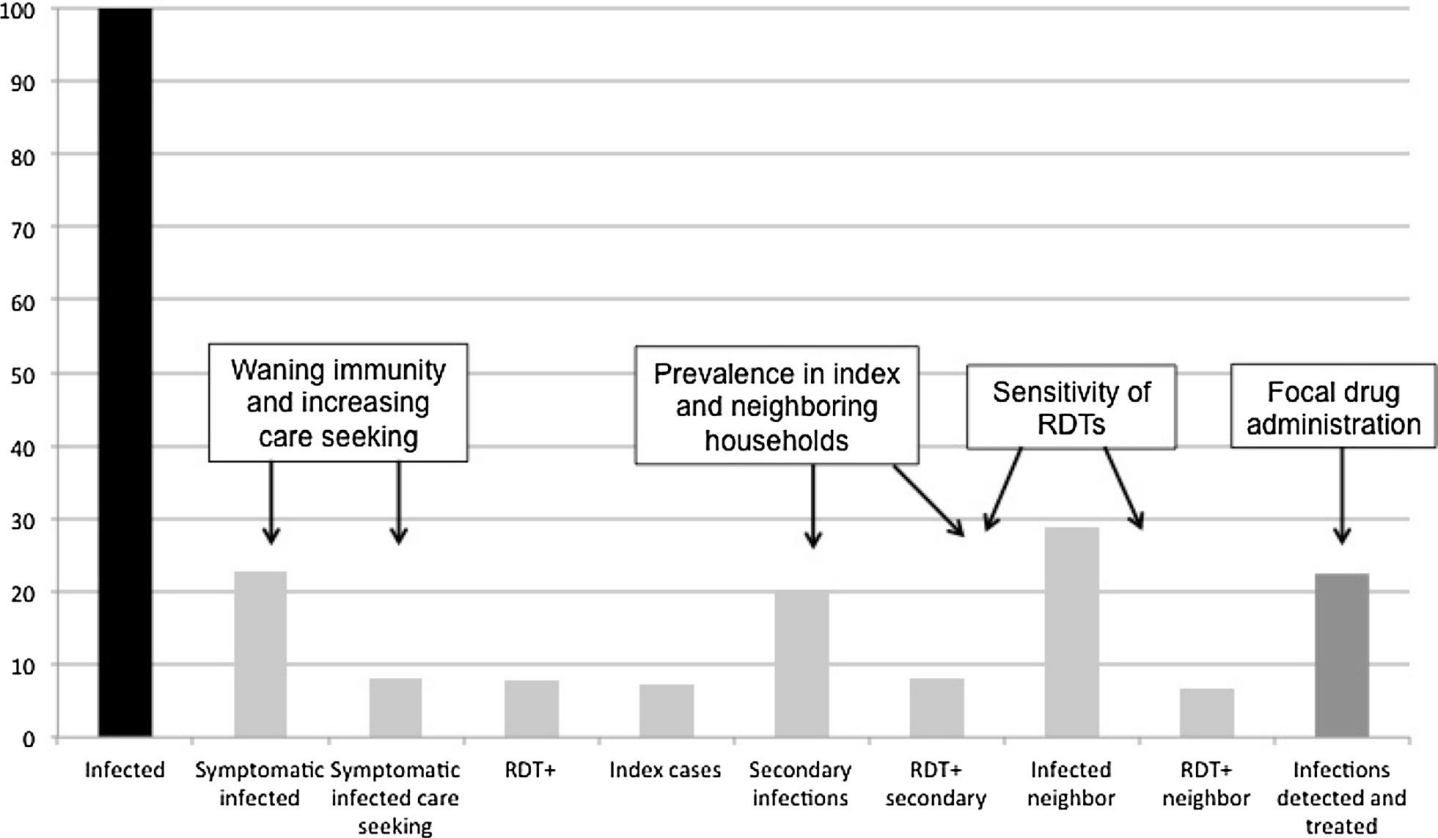
Key areas impacting the efficiency of reactive screen-and-treat on index and and neighbouring households

The cascades identified key areas that impact the effectiveness of reactive case detection to identify and treat infected individuals, including the ratio of symptomatic to asymptomatic infections, care-seeking behaviours and the RDT sensitivity (Fig. 8).

## Discussion

Implementation of reactive screen-and-treat in this area of southern Zambia faced several operational challenges, as would be expected with a new programme using volunteer CHWs and RDTs to expand clinical services into the community. Approximately one-third of eligible index case households resulted in reactive screen-and-treat and coverage decreased to one-quarter among RHCs with a higher burden of malaria. This low coverage was likely due to two factors. First, the follow-up screening was logistically difficult for CHWs due to the high number of cases during the peak malaria season. Step D activities were designed to be implemented when the number of malaria cases is approximately ten per week. Despite historically low transmission in this setting, some RHCs reported more than 70 eligible cases per month during the peak transmission season. This overwhelmed the capacity of the CHWs to conduct reactive case detection. During peak transmission times the programme would benefit from having additional CHWs available, or perhaps consider suspending reactive case detection during peak transmission.

The second challenge was insufficient RDTs as a consequence of the high number of cases and difficulty in anticipating the additional quantity of RDTs needed to conduct reactive case detection. Over 50 % of CHWs reported having at least 1 month when reactive screen-and-treat was not done due to lack of RDTs, and 40 % of CHWs reported at least 1 month when not even passive screening with RDTs could be performed. During this period, the parent RHCs did not report stock-outs or limited RDTs, implying delays, interruptions, or failures by RHC staff in distributing sufficient RDTs to the CHWs during the initial stages of implementing the reactive case detection programme. Following CHW training, a moderate stock of RDTs was provided to CHWs and RHC staff were notified that additional RDTs should be requested and released to CHWs to support Step D activities. Clearly to implement Step D, a reliable and ample supply of RDTs is necessary; however, the rapid seasonal changes in transmission makes predicting the required number of RDTs challenging and surge capacity may not be feasible. Over time, programmes should improve their ability to predict need and maintain an adequate stock of RDTs at the level of the RHPs. However, the cost may be that RDTs are stockpiled at these facilities and, if unused, may expire.

When eligible index cases were followed up, three main challenges were identified that hindered the ability to identify infected individuals through reactive screen-and-treat. First, only one-half to two-thirds of residents were at home at the time of screening and residents not at home were older than those at home. Those not at home included school-age children and young adults, the age group most commonly comprising the chronically infected reservoir that reactive case detection aims to identify and treat [3]. This challenge could be overcome. Notifications could be made to let individuals know when the CHW would be visiting. Households could be revisited to attempt to access those unable to be at home during the first visit. Second, while theoretically simple, identifying households within 140 m (the distance of one-and-a-half football fields) of an index case was difficult for the CHWs in practice. Sixty-eight per cent of neighbouring households screened by CHWs were outside the 140-m radius. Some CHWs screened neighbouring households over half a km from an index case household. While reactive screen-and-treat programmes in other countries screen further from the index household, in this setting nearly all RDT-positive individuals were within 250 m of the index household [6, 22, 36]. This demonstrates not only the difficulty in identifying the appropriate screening radius, but also the burden of unnecessary screening given the lack of RDTs and logistical challenges when the case burden is high. Lastly, as is widely recognized, the low sensitivity of RDTs limits the ability to identify individuals with low-level parasitaemia in areas approaching malaria elimination [14, 37, 38]. Even with complete follow-up, and with an RDT sensitivity of 40 % in index households and 23 % in neighbouring households, only 16 % of infected individuals were estimated to be identified by screening all index households and 22 % of infected individuals by screening neighbouring households. While the infectiousness of individuals with low parasitaemia is variable, a large portion of the malaria reservoir in this area would be not treated. Given the poor sensitivity of RDTs in this low-transmission setting, and the current lack of more sensitive field-deployable diagnostics, reactive focal drug administration may be a more efficient use of resources [17, 24]. With complete coverage, under the assumptions of the model, potentially 60 % of the total infected population in this setting would be treated through reactive focal drug administration of index and neighbouring households. However, complete coverage will be logistically difficult, and many improvements in follow-up strategies, including gauging neighbouring household distances, would need to be made [24].

A major limitation of this study was the short period of evaluation covering the rainy season but not the dry season. The study period (January to June 2014) only represents a six-month window where the evaluation was implemented. In addition, this period reflects early programme implementation. These limitations will likely underestimate the efficiency of the CHWs to react to incident cases as CHWs received continuous training and encouragement following the implementation of the programme and follow-up is easier during the dry season [9]. However, programmes implementing reactive case detection strategies can learn from this experience. These results highlight the need for monitoring and evaluation shortly after implementation to identify operational challenges and their potential impact on programme performance and impact early on.

A strength of the RHC survey is that demographic data on the number of residents not in the home at the time of screening were collected. The reactive screen-and-treat cascade used population-based survey data from the study area in Choma District. Information from Kalomo and Namwala Districts were not represented in these data; however, the people residing within the three districts are traditional subsistence farmers and are demographically similar. The model did not account for care seeking outside the government health facilities and assumed that all infected, symptomatic individuals sought care from government health facility, which may have overestimated the number of index cases detected through this system. However, the objective for creating the cascades was to provide estimates of the proportion of infected individuals identified and treated through reactive screen-and-treat and focal drug administration using multiple novel data sources.

## Conclusion

With limited resources, coverage and diagnostic tools, reactive screen-and-treat will likely not be sufficient to achieve malaria elimination in this setting. However, high coverage with reactive focal drug administration could be efficient at decreasing the reservoir of infection and should be considered as an alternative strategy.

### Additional files

**Additional file 1: FigS1.** Coverage cascades with complete coverage with malaria prevalence observed from data from Step D activities.

**Additional file 2: FigS2.** Coverage cascades with complete coverage with malaria prevalence from RHP evaluation and RDT sensitivity doubled.

**Additional file 3: FigS3.** Coverage cascades with complete coverage with malaria prevalence from RHP evaluation and symptomatic infections doubled.
