# Supplementary figures and images for: Evaluation of the operational challenges in implementing reactive screen-and-treat and implications of reactive case detection strategies for malaria elimination in a region of low transmission in southern Zambia
Source: Malar J. 2016 Aug 15;15:412. doi: 10.1186/s12936-016-1460-x (PMC4986207; doi:10.1186/s12936-016-1460-x)

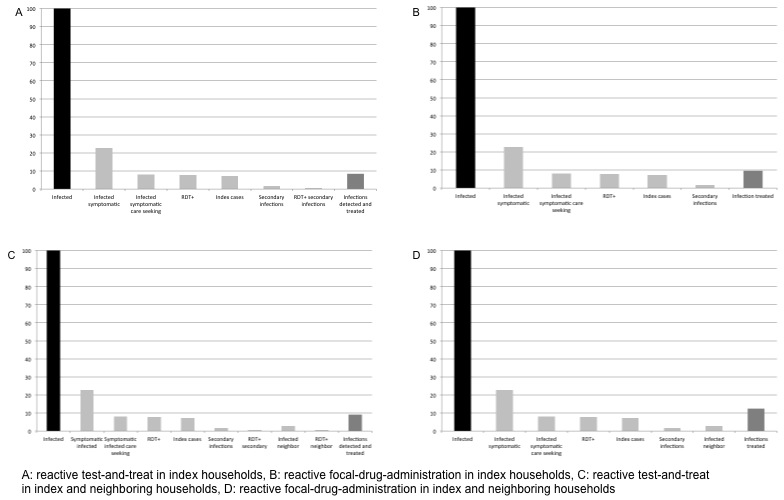

Supplement: Supplementary file 1 — Additional file 1: FigS1. Coverage cascades with complete coverage with malaria prevalence observed from data from Step D activities. [file 12936_2016_1460_MOESM1_ESM.jpg]

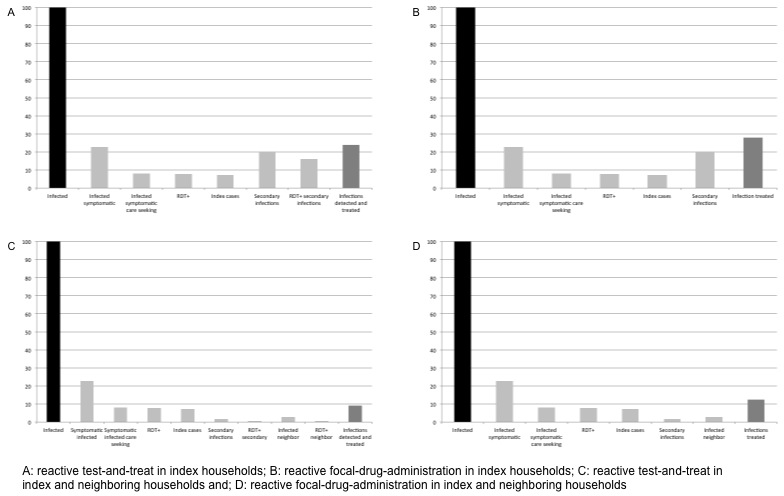

Supplement: Supplementary file 2 — Additional file 2: FigS2. Coverage cascades with complete coverage with malaria prevalence from RHP evaluation and RDT sensitivity doubled. [file 12936_2016_1460_MOESM2_ESM.jpg]

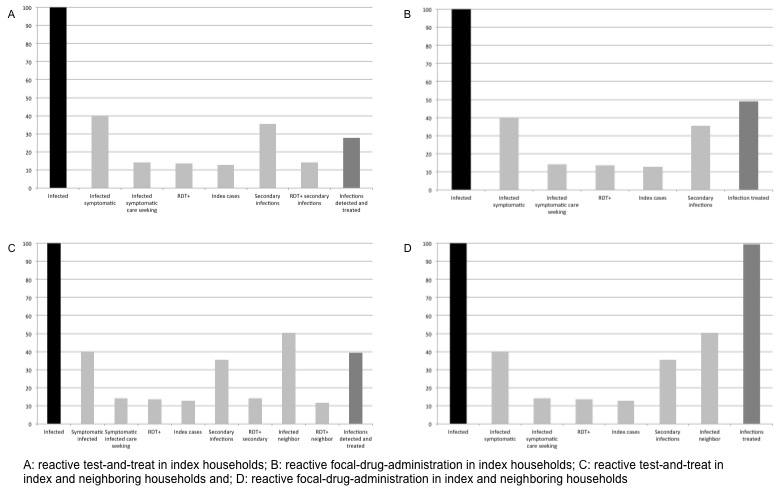

Supplement: Supplementary file 3 — Additional file 3: FigS3. Coverage cascades with complete coverage with malaria prevalence from RHP evaluation and symptomatic infections doubled. [file 12936_2016_1460_MOESM3_ESM.jpg]
